# External validation of the Oncotype DX breast cancer recurrence score nomogram and development and validation of a novel machine learning-based model to predict postoperative overall survival and guide adjuvant chemotherapy in ER positive, Her-2 negative breast cancer patients: a retrospective cohort study

**DOI:** 10.3389/fonc.2025.1586262

**Published:** 2025-05-21

**Authors:** Dongdong Wang, Xinfeng Wang, Xin Yang

**Affiliations:** ^1^ Department of General Surgery, Beijing Hospital, National Center of Gerontology, Institute of Geriatric Medicine, Chinese Academy of Medical Sciences, Beijing, China; ^2^ Department of General Surgery, Beijing Friendship Hospital, Capital Medical University, Beijing, China

**Keywords:** breast cancer, machine learning, prognostic model, guidance for adjuvant chemotherapy, web calculator

## Abstract

**Background:**

This study aims to externally validate the performance of the Oncotype DX (ODX) breast cancer (BC) recurrence score nomogram in predicting adjuvant chemotherapy (ACT) for BC after surgery and subsequently develop a machine learning-based model to predict postoperative overall survival (OS) and guide ACT, demonstrating superior comprehensive performance.

**Methods:**

This analysis leveraged data from the SEER database spanning 2010-2020, alongside a BC cohort from the Beijing Hospital (BJH). Machine learning methods were applied for predictor selection by wrapper methods and the development of the predictive model. The optimal model was determined using the concordance index (C-index), time-dependent calibration curves, time dependent receiver operating characteristic (ROC) curves, and decision curve analysis (DCA). The benefit analysis of ACT was primarily conducted using Kaplan-Meier survival analysis.

**Results:**

The ODX nomogram performed poorly in predicting ACT benefit in both the SEER cohort and the BJH cohort. Subsequently, we employed ten machine learning methods to develop ten prognostic models. The Accelerated oblique random survival forest model (AORSFM), exhibiting the highest prediction performance, was selected. The C-index for AORSFM is 0.799 (95% CI 0.779-0.823) in the SEER cohort and 0.793 (95% CI 0.687-0.934) in the BJH cohort. Furthermore, time-dependent calibration curves, time-dependent ROC analysis, and DCA indicate that the AORSFM demonstrates good calibration, predictive accuracy, and clinical net benefit. A publicly accessible web tool was developed for the AORSFM. Notably, the new staging system based on AORSFM can provide guidance for postoperative ACT in such patients.

**Conclusions:**

The AORSF has the potential to identify postoperative OS and guide ACT in patients with BC. This can assist clinicians in assessing the severity of the disease, facilitating patient follow-up, and aiding in the formulation of adjuvant treatment strategies.

## Introduction

Breast cancer (BC) is one of the most common malignant tumors among women worldwide, ranking as the second most prevalent cancer in females and the leading cause of cancer-related mortality in women (1, 2). Postoperative adjuvant chemotherapy (ACT) is a crucial component of comprehensive BC treatment, significantly reducing the risk of recurrence and improving patient survival (3, 4). However, its efficacy varies among individuals, with some patients deriving only minimal survival benefits while experiencing chemotherapy-related toxicity (5).

Orucevic et al. developed a nomogram—the Oncotype DX (ODX) nomogram—as a cost-effective alternative to the expensive ODX breast cancer recurrence score, which is based on a 21-gene assay (6, 7). It can be used for treatment decision-making in hormone receptor-positive, Human Epidermal Growth Factor Receptor 2 (Her-2)-negative BC patients. However, the predictive capability of the ODX nomogram for the efficacy of ACT lacks sufficient external validation, leading to a lack of reliable predictive tools to guide ACT. This issue urgently needs to be addressed.

Previously, Fihn et al. and Markowetz et al. provided several standards for researchers developing predictive models to make clinical prediction models more “useful.” (8, 9) The key points include: 1. Simplicity in obtaining input features: The input features should be easy to acquire, ideally commonly found in routine clinical diagnostics. 2. Clinically relevant outputs: The output parameters should aid in clinical decision-making. 3. Ease of application: The model should be simple and easy to use. Given these criteria, it seems that all models based on biological prognostic markers are not currently considered “useful” models.

Recently, with the growing popularity of machine learning methods, machine learning-based predictive models have gained significant traction in oncology research. For instance: A study developed a recurrence prediction model for duodenal adenocarcinoma using basic clinical features, which demonstrated moderate predictive performance and outperformed AJCC staging in predicting postoperative recurrence (10). Another study employing bioinformatics analysis developed a long non-coding RNA (lncRNA)-based machine learning model for colorectal cancer prognosis, which also demonstrated outstanding predictive performance (11). The key advantage of models based on basic clinical features lies in their: Accessibility, Generalizability, and Cost-effectiveness. In contrast, models relying on transcriptomic markers like lncRNAs lack these practical advantages for clinical implementation. However, there remains a notable gap in the field: no existing predictive model utilizing basic clinical features has been specifically developed for forecasting ACT efficacy in BC patients.

Therefore, this study aims to externally validate the ODX nomogram using BC patient data from the SEER database and Beijing Hospital (BJH) ‘s BC cohort. Additionally, the study seek to develop and validate a machine learning model to predict postoperative overall survival (OS) in BC patients and guide ACT.

The main novelty of this study lies in two key contributions: First, the study conducted the first external validation of the ODX nomogram, specifically evaluating its performance in predicting ACT benefit (rather than recurrence risk). Second, recognizing the limited predictive capability of the ODX nomogram for chemotherapy response, the study developed and validated a novel prediction model specifically designed for this purpose. This new model incorporates the same clinical features used in the ODX nomogram (tumor size, age, grade, progesterone receptor (PR) status, and histopathology) but is optimized for chemotherapy efficacy prediction.

## Materials and methods

**Figure 1:**
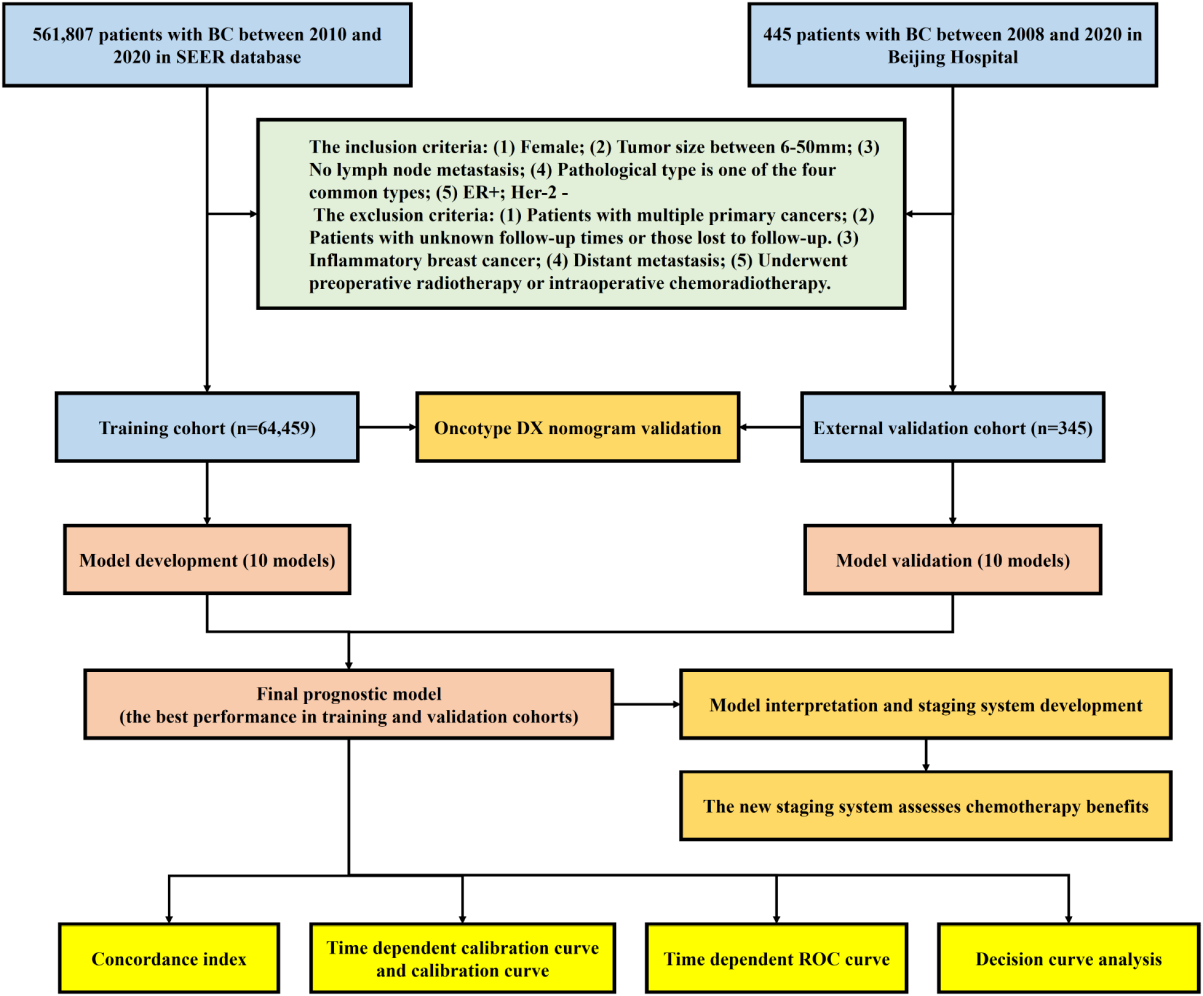
Flow diagram of the study.

This study strictly adhered to the Prediction model Risk Of Bias Assessment Tool (PROBAST) standards and a checklist for useful clinical prediction tools reported by Florian Markowetz, and followed the Transparent Reporting of a Multivariable Prediction Model for Individual Prognosis or Diagnosis (TRIPOD) Checklist for reporting (9, 12, 13). The complete study process of this study is shown in **Figure 1**.

### Study population

This retrospective cohort study included patients diagnosed with BC from the SEER database between 2010 and 2020 and Beijing Hospital between 2008-2020. Prior to the subsequent follow-up survey, each patient provided informed consent. Surgical procedures were conducted by expert surgeons. Adherence to ethical standards was ensured by conducting the study in compliance with the Declaration of Helsinki (revised in 2013), and ethical approval was obtained from the Hospital Ethics Committee of the BJH (2022BJYYEC-360-01). Patients diagnosed with BC in the SEER database (SEER cohort) were assigned to the training cohort, while patients diagnosed with BC at BJH (BJH cohort) were assigned to the external validation cohort.

### Inclusion and exclusion criteria

The inclusion criteria were as follows: (1) Female; (2) Tumor size between 6-50mm; (3) No lymph node metastasis; (4) Pathological type is one of the four common types (Invasive Ductal Carcinoma (IDC), Invasive Lobular Carcinoma (ILC), IDC+ILC, and IDC+Other type); (5) Estrogen Receptor positive (ER+); Human Epidermal Growth Factor Receptor 2 negative.

The exclusion criteria were as follows: (1) Patients with multiple primary cancers; (2) Patients with unknown follow-up times or those lost to follow-up. (3) Inflammatory breast cancer; (4) Distant metastasis; (5) Underwent preoperative radiotherapy or intraoperative chemoradiotherapy.

A total of 561,807 patients with BC were identified. Among them, 497,003 patients who did not meet the inclusion criteria and were excluded. Consequently, 64,804 patients were included in the analysis.

### Survival analysis and subgroup analysis

Patients from the SEER and BJH cohorts were classified into three risk groups—high, intermediate, and low—based on the ODX nomogram or novel machine learning-based model. The study then conducted survival analysis for each of the three risk groups within both cohorts. The Kaplan-Meier (KM) survival curves and log-rank test were utilized to assess differences in survival rate for each risk group and each chemotherapy group. Subgroup analysis was conducted to assess the chemotherapy benefit among high-risk, intermediate-risk, and low-risk groups of patients in the SEER and BJH cohorts. Given the multiple comparisons, the study applied the Bonferroni correction method to adjust the *p*-values in the subgroup analyses, thereby reducing the incidence of Type I errors.

### Development and validation of novel machine learning model

The Four variables in the ODX predictive nomogram of the training cohort, including Tumor size, age, grade, PR status, and histopathology were included in ten machine learning models to develop novel predictive models.

The study chose widely recognized machine learning models capable of handling both continuous and categorical variables. These included: Akritas Estimator (AKE), Gradient Boosting (GB), Generalized Additive Models via Gradient Boosting (GAMB), Generalized Linear Models via Gradient Boosting (GLMB), Survival Tree (ST), Conditional Inference Tree (CIT), Random Survival Forest (RSF), Conditional Random Forest (CRF), Accelerated Oblique Random Survival Forest (AORSF), Penalized Regression method (PRM). These models were all sourced from the “mlr3proba” R package (14).

The AKE is a non-parametric estimation method used in regression analysis to handle censored or truncated dependent variables. It estimates the relationship between the dependent and independent variables through ranking and regression techniques (15). GB is an ensemble learning algorithm that iteratively adds weak learners (typically decision trees) to fit the residuals of the previous step, progressively optimizing the loss function to enhance the overall predictive performance of the model (16). The GAMB and GLMB are ensemble learning methods that enhance the predictive performance and adaptability of a model by iteratively adding and adjusting a series of base learners from Generalized Additive Models or Generalized Linear Model (17). ST is a decision tree model used in survival analysis that recursively splits data to group individuals based on their survival times and the risk of events occurrence, thereby providing insights into survival probabilities (18). Besides, ST can also be used to identify a cut-off value. The CIT is a method for constructing decision trees that uses statistical tests to select splitting variables and points, thereby reducing bias and overfitting in the model during the splitting process in a non-parametric and conditional inference-based manner (19). RSF is an ensemble learning method used in survival analysis that constructs multiple survival trees and uses the results from these trees to estimate the survival functions and hazard ratios for individuals, thereby offering powerful analysis of survival time data (20). The CRF is an ensemble learning algorithm that builds multiple decision trees and uses conditional inference tests to select variables and splitting points. This approach enhances the robustness and accuracy of the model while reducing bias in variable selection (21). The AORSF is a survival analysis model that combines the ensemble learning techniques of random forests with the approach of oblique decision trees. This integration enhances the accuracy and efficiency in handling complex survival data (22). PRM is a regression analysis technique that controls model complexity by adding penalty terms (such as L1 or L2 regularization) to the loss function. This approach helps to prevent overfitting and enhances the model’s generalization ability (23).

The ten machine learning learners were used to develop models. These models were trained using the training cohort, culminating in 10 prediction models. These models were subsequently validated within the external validation cohort.

The C-index was utilized to assess the models performance. As a statistical measure for evaluating the predictive capability of survival analysis models, the C-index is widely used in medical study. It gauges the congruence between model predictions and actual outcomes, with its value ranging from 0 to 1. A higher C-index indicates superior predictive accuracy of the model. The model with the highest average C-index of two cohorts was chosen for further investigation. Time-dependent calibration curves were used to reflect the degree of calibration over the entire time range. Further, the calibration curves were generated to evaluate the correspondence between predicted and actual survival rates of dead at 1, 3, 5, and 10 years. The area under the time-dependent receiver operating characteristic (ROC) curves (AUC) served to compare the predictive accuracy and discriminative power of the model and its components. Decision curve analysis (DCA) was conducted to determine the clinical utility of the model, assessing the clinical benefits for patients at 1, 3, 5, and 10 years. Risk scores were calculated in the training and validation cohorts using the selected machine learning model.

To examine how different features influence model performance over time, a time-dependent feature importance analysis method was employed. The significance of each predictor was evaluated by computing the model’s Brier score loss after permuting feature values, with this process repeated through a 10-fold cross-validation resampling strategy for statistical reliability. This approach enabled identification of which features’ importance for model predictions varies over time, providing insights crucial for time-sensitive clinical decision-making.

### Development of a web risk calculator and a staging system

The ST was employed to determine the cut-off value for the risk score, thereby classifying patients into high-risk, medium-risk, and low-risk group. Furthermore, a web-based application was developed to make these predictive models accessible online, utilizing the R package “shiny” for its development (24).

### Statistical analysis

Kolmogorov-smirnov test was used to assess whether the data followed a normal distribution. For normally-distributed continuous variables, the data were described as mean ± standard deviation and compared using the t-test. If continuous variables did not conform to a normal distribution, the MannWhitney U test was used, and results were presented as median (interquartile range). Categorical data were presented as numbers and frequencies, and either the Chi-square test or Fisher’s exact test was used for comparisons. All statistical tests were two-sided, with P-values and Bonferroni-adjusted P-value <0.05 indicating statistical significance. All figure illustrations and statistical analyses were conducted using R version 4.4.1.

## Results

### Patient characteristics

The baseline characteristics of the SEER cohort (training cohort, n = 64,459) and BJH cohort (external validation cohort, n = 345) are presented in **Table 1**. In the SEER cohort, the median age is 67 and the median follow-up time is 17 months. In the BJH cohort, the median age is 65 and the median follow-up time is 18 months.

**Table 1:** Baseline characteristics of SEER and BJH cohorts.

Feature	SEER cohort (n=64,459)	BJH cohort (n=345)	*P* value
Age (years)	64.0 [52.0;74.0]	59.0 [49.0;67.0]	<0.001
Tumor size (mm)	15.0 [10.0;22.0]	20.0 [15.0;25.0]	<0.001
T stage			<0.001
T1b	16928 (26.3%)	41 (11.9%)	
T1c	29386 (45.6%)	176 (51.0%)	
T2	18145 (28.1%)	128 (37.1%)	
Pathological type			<0.001
IDC	49122 (76.2%)	265 (76.8%)	
IDC+ILC	2295 (3.56%)	4 (1.16%)	
IDC+Other	4591 (7.12%)	61 (17.7%)	
ILC	8451 (13.1%)	15 (4.35%)	
Grade			<0.001
Grade 1	20505 (31.8%)	69 (20.0%)	
Grade 2	33576 (52.1%)	221 (64.1%)	
Grade 3	10378 (16.1%)	55 (15.9%)	
PR			0.845
Negative	6981 (10.8%)	39 (11.3%)	
Positive	57478 (89.2%)	306 (88.7%)	
Adjuvant chemotherapy			0.129
No	22425 (34.8%)	134 (38.8%)	
Yes	42034 (65.2%)	211 (61.2%)	
Survival status			<0.001
Alive	56987 (88.4%)	333 (96.5%)	
Dead	7472 (11.6%)	12 (3.48%)	
Follow-up time (months)	54.0 [26.0;87.0]	80.0 [56.8;102]	<0.001

### Validation of the ODX breast cancer recurrence score nomogram

**Figure 2:**
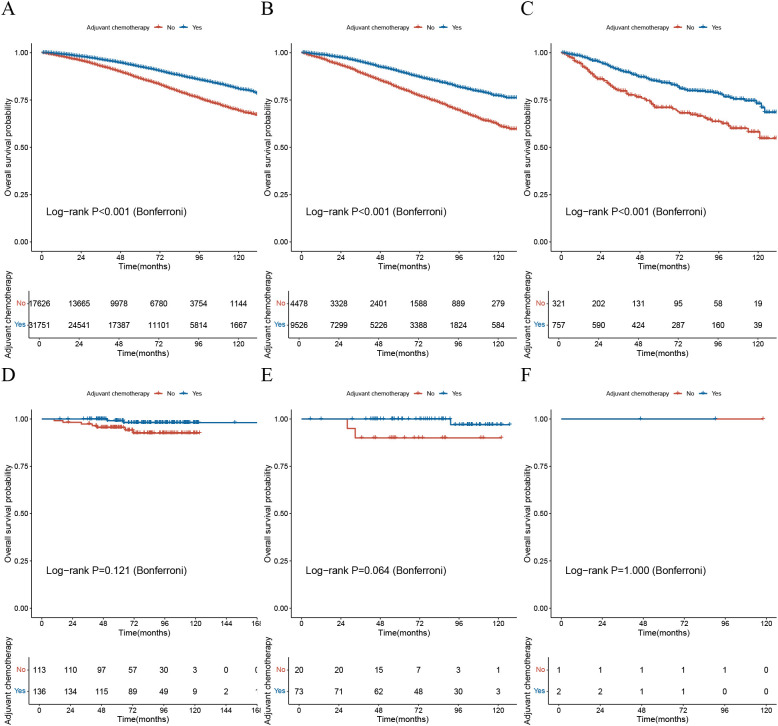
Validation of the Oncotype DX nomogram in assessing chemotherapy benefit. **(A)** The KM survival curve for the low-risk group based on the ODX nomogram in the SEER cohort; **(B)** The KM survival curve for the intermediate-risk group based on the ODX nomogram in the SEER cohort; **(C)** The KM survival curve for the high-risk group based on the ODX nomogram in the SEER cohort; **(D)** The KM survival curve for the low-risk group based on the ODX nomogram in the BJH cohort; **(E)** The KM survival curve for the intermediate-risk group based on the ODX nomogram in the BJH cohort; **(F)** The KM survival curve for the high-risk group based on the ODX nomogram in the BJH cohort; KM, Kaplan-Meier; ODX, Oncotype DX; BJH, Beijing Hospital.

Based on the nomogram formula provided in the literature (7), the study calculated the nomogram scores and predicted probabilities for all patients in both cohorts. According to the standards outlined in the study, patients were classified into high, intermediate, and low-risk groups, and survival analysis was conducted to assess the chemotherapy benefit across different risk groups (**Figure 2**).

The results indicated that in the SEER cohort, patients in all three risk groups who received adjuvant chemotherapy had significantly better OS compared to those who did not, with statistical significance (*p*-value<0.001) (**Figures 2A–C**). However, in the BJH cohort, there was no statistically significant difference in OS between patients who received adjuvant chemotherapy and those who did not, across all three risk groups (*p*-value>0.05) (**Figures 2C–E**).

These findings suggest that the ODX nomogram may not accurately assess the benefit of chemotherapy.

### Model development, validation, and evaluation

For the development of model using the training cohort, five variables were processed through ten machine learning learners and subsequently validated across the external validation cohort. This process resulted in a total of ten machine learning models specifically designed for predicting OS.

**Figure 3:**
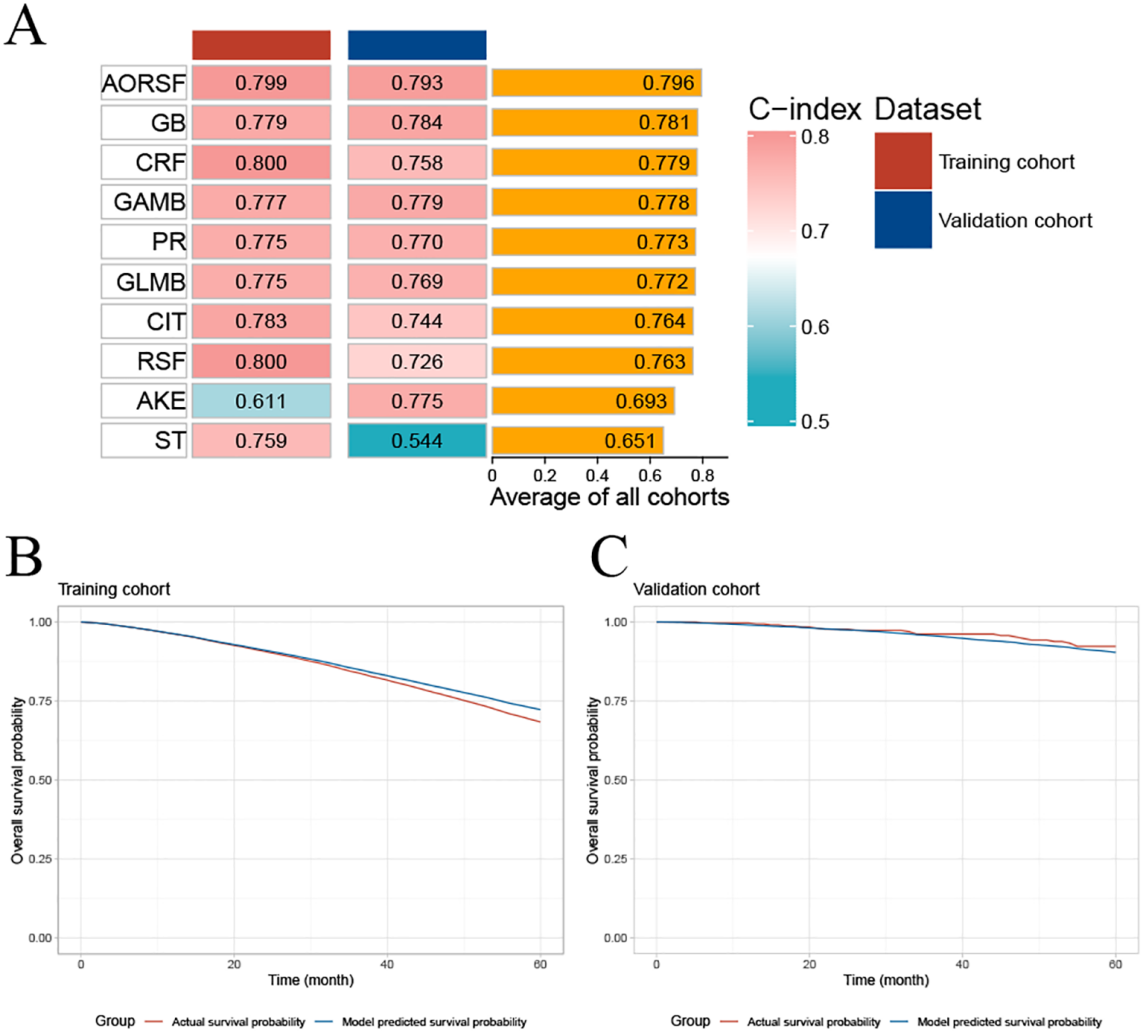
Concordance index of ten machine learning models and evaluating the calibration of AORSFM by time-dependent calibration curves. **(A)** The C-index for the ten machine learning models was calculated for the training cohort and validation cohort. Ranking of the models was based on the average C-index of two cohorts; **(B)** Time-dependent calibration curve in training cohort; **(C)** Time-dependent calibration curve in validation cohort. AKE, Akritas estimator; GB, Gradient Boosting, GAMB, Boosted Generalized Additive Model; GLMB, Boosted Generalized Linear Model; ST, Survival Tree; CIT, Conditional Inference Tree; RSF, Random Survival Forest; CRF, Conditional Random Forest; AORSF, Accelerated Oblique Random Survival Forest; PR, Penalized Regression Method; C-index, concordance index; AORSFM, Accelerated Oblique Random Survival Forest Model.

Initial evaluation focused on the C-index, with rankings C-indexes of all ten prediction models displayed in **Figure 3A**. The AORSF model (AORSFM) showcased the highest average C-index at 0.796 in training and validation cohorts, making it the most effective model among all. The C-index for AORSFM was 0.799 (95% CI 0.779-0.823) in the training cohort and 0.793 (95% CI 0.687-0.934) in the validation cohort, marking the highest values compared to other models.

**Figure 4:**
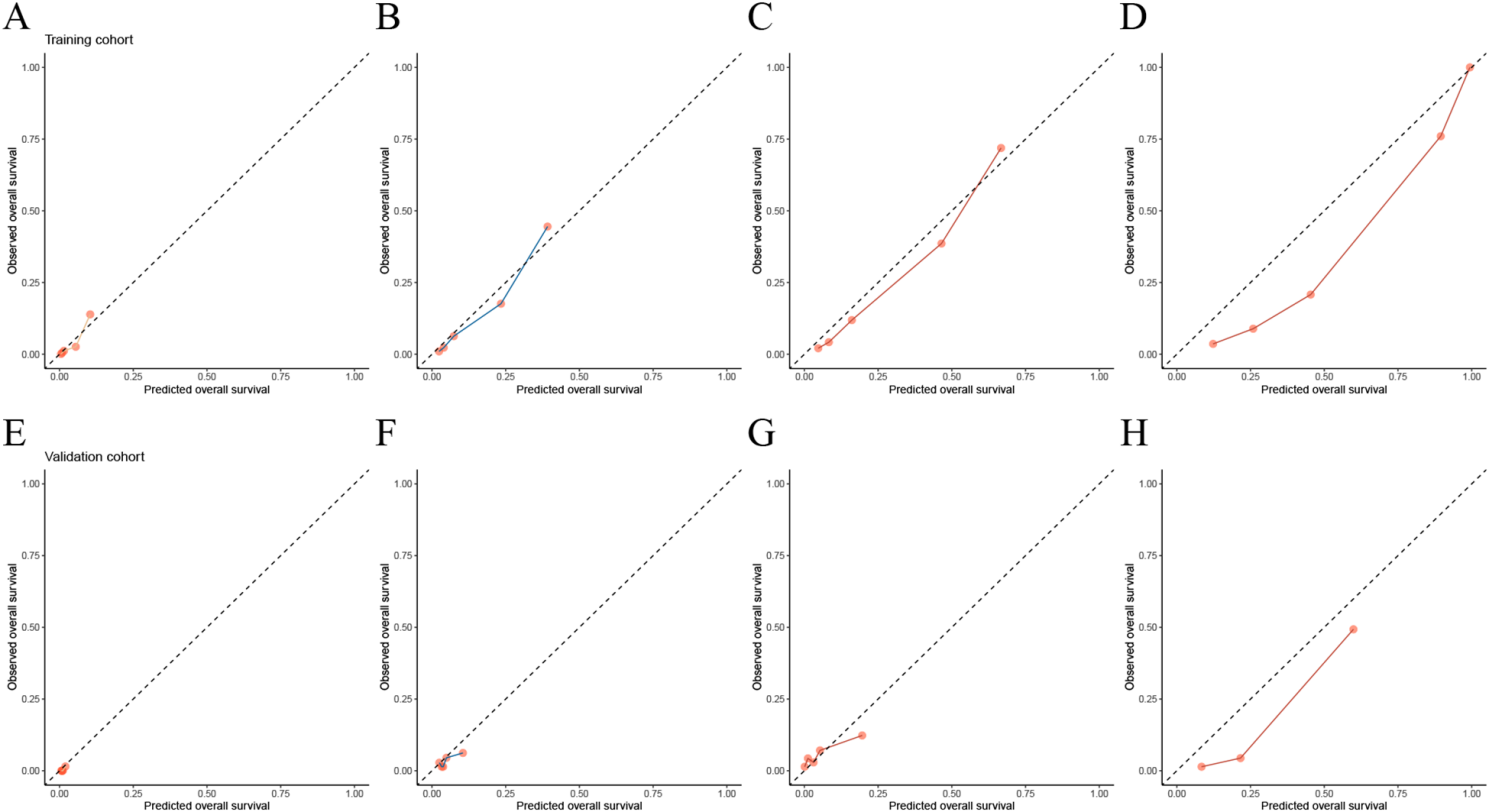
Evaluating the calibration of AORSFM by calibration curves. **(A)** Calibration curves in 1-year in training cohort, **(B)** Calibration curves in 3-year in training cohort, **(C)** Calibration curves in 5-year in training cohort, **(D)** Calibration curves in 10-year in training cohort, **(E)** Calibration curves in 1-year in validation cohort, **(F)** Calibration curves in 3-year in validation cohort, **(G)** Calibration curves in 5-year in validation cohort, **(H)** Calibration curves in 10-year in validation cohort.

The time-dependent calibration curves, along with calibration curves for the training cohort and validation cohort at 1, 3, 5, and 10 years, demonstrate that AORSFM achieved good calibration in the training cohort and validation cohort. (**Figures 3B, C** and **Figures 4A–H**).

The AUC demonstrate the strong predictive accuracy of AORSFM over 1, 3, 5, and 10 years in the training cohort and validation cohort (**Table 2**).

**Table 2:** The AUC and confidence intervals (CI) of AORSFM for the training and validation cohorts at 1, 3, 5, and 10 years.

Cohort	AUC at 1 year (95%CI)	AUC at 3 years (95%CI)	AUC at 5 years (95%CI)	AUC at 10 years (95%CI)
Training cohort	0.795 (0.780-0.808)	0.821 (0.813-0.828)	0.824 (0.819-0.829)	0.843 (0.834-0.851)
Validation cohort	0.975 (0.958-0.992)	0.955 (0.904-1.000)	0.938 (0.852-0.991)	0.842 (0.534-1.000)

**Figure 5:**
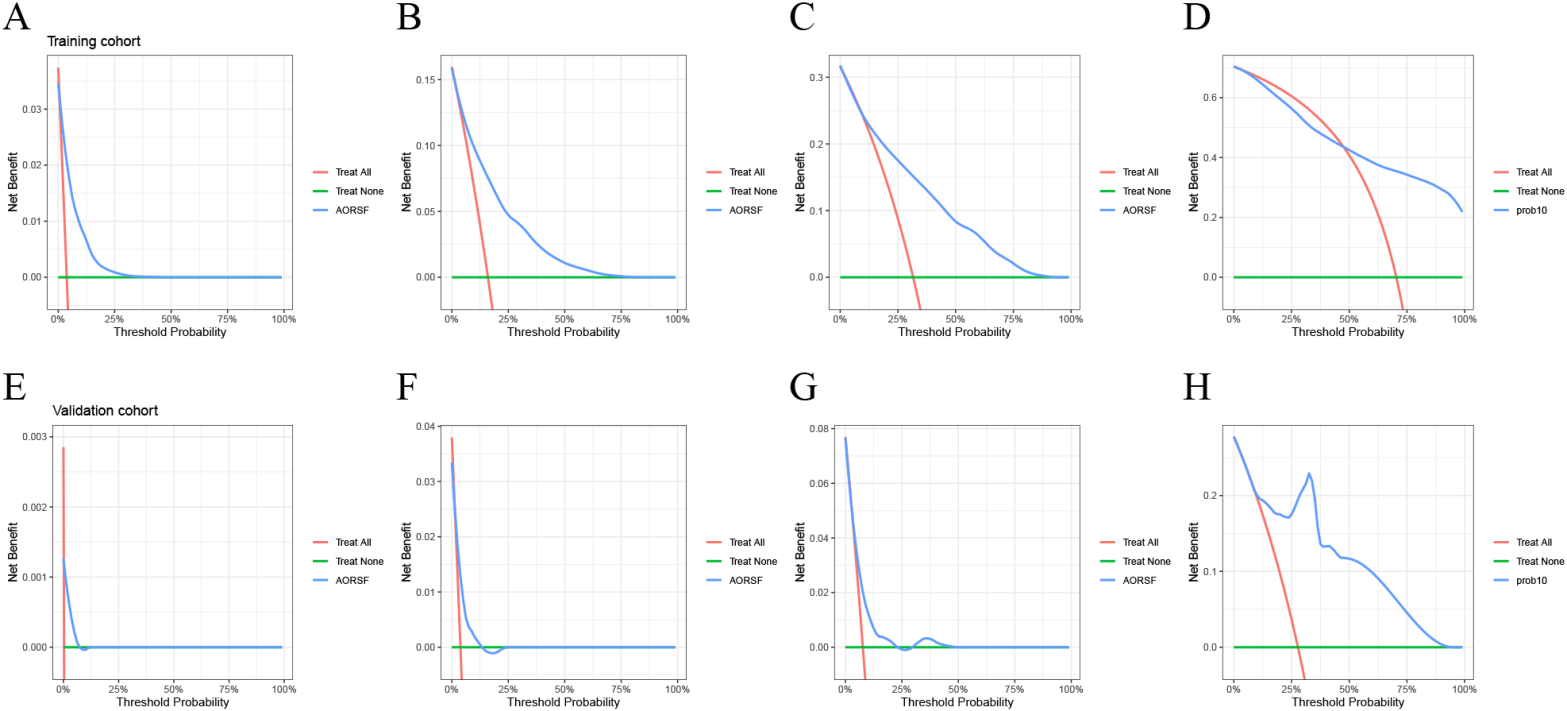
Evaluating the net benefit of AORSFM by DCA. **(A)** 1-year DCA for training cohort, **(B)** 3-year DCA for training cohort, **(C)** 5-year DCA for training cohort, **(D)** 10-year DCA for training cohort, **(A)** 1-year DCA for validation cohort, **(B)** 3-year DCA for validation cohort, **(C)** 5-year DCA for validation cohort, **(D)** 10-year DCA for validation cohort. DCA, Decision curve analysis.

The DCA for the AORSFM demonstrated a consistent net benefit in the training cohort and validation cohort over a range of threshold probabilities. (**Figure 5**) In two cohorts, the AORSFM outperformed the ‘treat none’ and ‘treat all’ strategies, indicating that it had practical utility in decision-making.

### Model interpretation and development of new stage system

**Figure 6:**
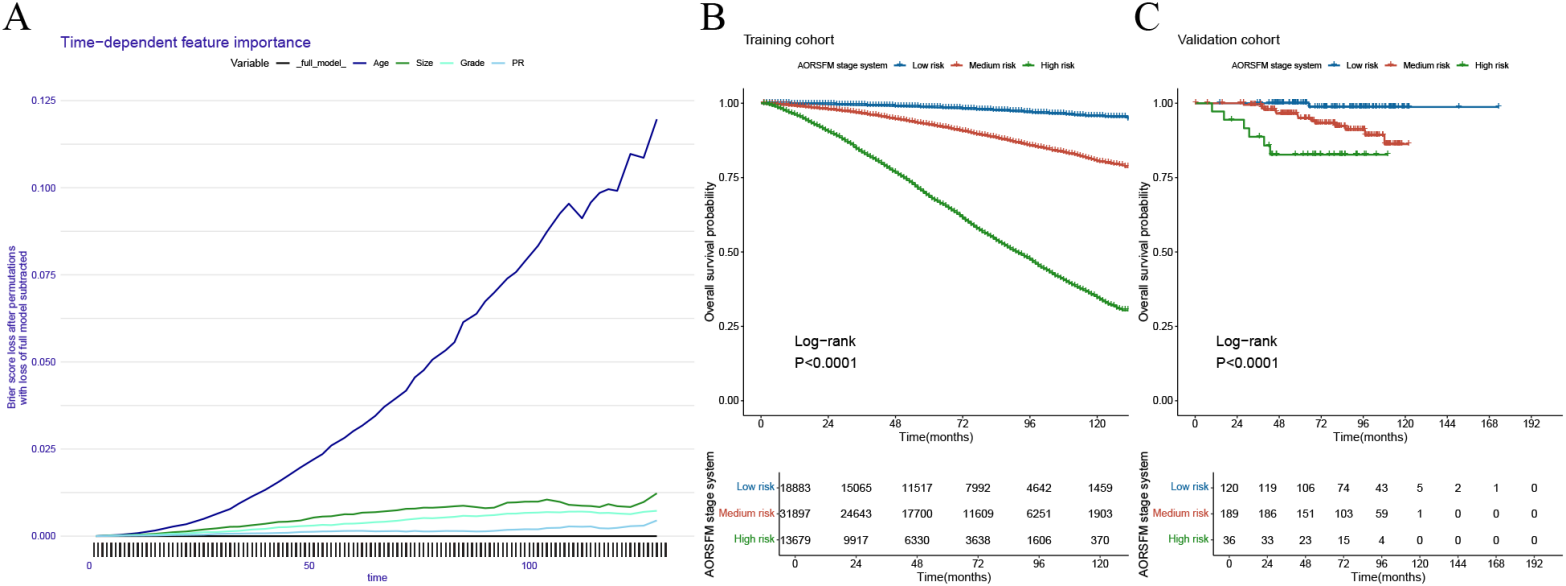
Interpretation the AORSF by time-dependent feature importance curves and the performance of the AORSFM staging system in the training cohort and validation cohort. **(A)** The time-dependent feature importance curves of AORSFM; **(B)** The KM survival curves for the AORSFM staging system in the training cohort, **(C)** The KM survival curves for the AORSFM staging system in the validation cohort. AORSFM, Accelerated Oblique Random Survival Forest Model; KM, Kaplan-Meier.

The time-dependent feature importance curves show the varying importance of each predictor in AORSFM over time. (**Figure 6A**) The results indicate that apart from the increasing importance of “age” over time, the importance of other parameters remained stable. To further enhance the usability of the model, the study developed a web-based risk calculator. (https://pgsxuliu.shinyapps.io/AORSFMforBC/) Additionally, the study developed a staging system based on AORSFM, utilizing the ST learner to categorize BC patients into three risk groups according to AORSFM’s risk scores. KM survival curves demonstrate statistically significant differences in OS among the high-risk, medium-risk, and low-risk groups across the two cohorts. (**Figures 6B-C**).

### The significance of the AORSFM stage system in guiding postoperative chemotherapy

**Figure 7:**
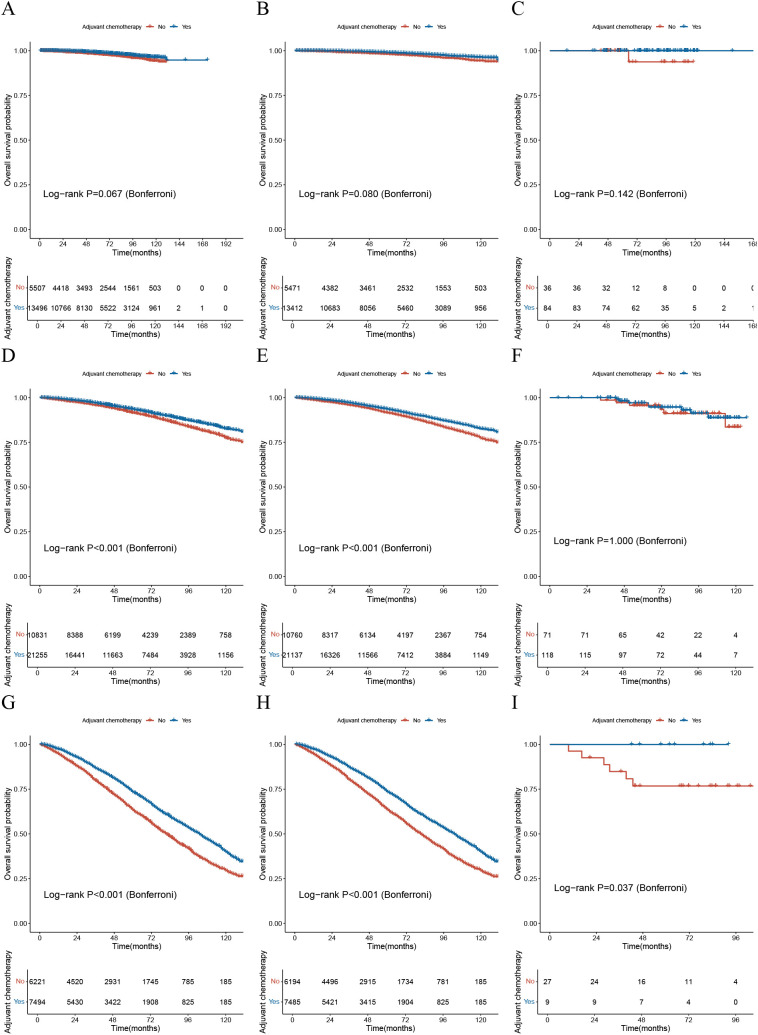
The significance of the AORSFM stage system in guiding postoperative chemotherapy. **(A)** The KM survival curve for the low-risk group based on the AORSFM stage system in the total cohort; **(B)** The KM survival curve for the low-risk group based on the AORSFM stage system in the training cohort; **(C)** The KM survival curve for the low-risk group based on the AORSFM stage system in the validation cohort; **(D)** The KM survival curve for the intermediate-risk group based on the AORSFM stage system in the total cohort; **(E)** The KM survival curve for the intermediate-risk group based on the AORSFM stage system in the training cohort; **(F)** The KM survival curve for the intermediate-risk group based on the AORSFM stage system in the validation cohort; **(G)** The KM survival curve for the high-risk group based on the AORSFM stage system in the total cohort; **(H)** The KM survival curve for the high-risk group based on the AORSFM stage system in the training cohort; **(I)** The KM survival curve for the high-risk group based on the AORSFM stage system in the validation cohort. KM, Kaplan-Meier; AORSFM, Accelerated Oblique Random Survival Forest Model.

The study plotted survival curves based on the high, intermediate, and low-risk groups defined by the AORSFM stage system in both cohorts to evaluate the survival differences between the postoperative chemotherapy group and the non-chemotherapy group. The results indicated that in the total cohort, the training cohort, and the validation cohort, there was a trend of difference in OS between patients in the low-risk group who received chemotherapy and those who did not, but this difference was not statistically significant (*p*-value>0.05 in total, training and validation cohorts) (**Figures 7A-C**). In the intermediate-risk group, the total cohort and training cohort exhibited survival differences between the chemotherapy and non-chemotherapy groups, which differed from the validation cohort (*p*-value<0.001 in total and training cohorts; *p*-value>0.05 in validation cohort) (**Figures 7D-F**). However, in the high-risk groups, there was a significant difference in OS between patients who received chemotherapy and those who did not, with statistical significance (*p*-value<0.05 in total, training and validation cohorts) (**Figures 7G-I**). This suggests that the AORSFM stage system can effectively assess the benefit of postoperative chemotherapy and provide guidance for postoperative chemotherapy in patients.

## Discussion

In this study, the study externally validate the ODX nomogram in SEER and a single-center BC cohort. The results seem to be unsatisfactory, as the risk groups defined by the ODX nomogram in both cohorts do not effectively guide postoperative chemotherapy decisions for patients.

Therefore, the study developed and validated a novel machine learning-based model to accurately predict postoperative OS in patients with BC and to provide guidance for postoperative chemotherapy decisions. The prognostic model exhibited accuracy in the large training cohort and external validation cohort. In terms of predictive values, AORSFM generally exhibits a high C-index and AUC, indicating the model’s accuracy and stability in predicting patients’ OS. Additionally, the time-dependent calibration curves and DCA demonstrate the excellent calibration and clinical net benefit of AORSFM. This study indicates that AORSFM has the potential to identify OS in BC patients and can provide guidance for postoperative chemotherapy decisions. This can assist clinicians in assessing the severity of the disease, facilitating patient follow-up, and aiding in the formulation of adjuvant treatment strategies.

Recently, the development of predictive models has gained significant attention among clinical scientists. Therefore, the standardized development and validation of predictive models are crucial, and this study adhered rigorously to these standards. Finhn et al. noted that the proliferation of predictive models has been accompanied by an increasing awareness of the need for standards to ensure their accuracy. A significant milestone was the publication of the TRIPOD guidelines nearly a decade ago (8, 13). Wolff et al. developed a tool to assess the risk of bias and the applicability of prediction model studies (12). This tool includes 20 signal questions designed to enable researchers to self-assess their studies. Florian Markowetz proposed a checklist for useful clinical prediction tools aimed at making clinical prediction models impactful for patients (9). The aforementioned checklist and tools were used to standardize this study.

The ODX assay and its accompanying nomogram represented a seminal advancement in personalized oncology by stratifying recurrence risk and predicting adjuvant chemotherapy benefit through genomic profiling (6, 7, 25). While the chemotherapy response prediction function of the ODX nomogram was conceptually innovative, the external validation reveals its limited discriminative capacity in actual clinical decision-making. This critical shortfall in identifying true chemotherapy responders versus non-responders motivated our development of AORSFM. AORSFM addresses a critical unmet need in contemporary chemotherapy decision-making by providing dynamic prognostic stratification. Clinicians could leverage this adaptive tool to identify patients likely to derive survival benefits from specific chemotherapy regimens while sparing others from unnecessary toxicity (26). Particularly for the hormone receptor-positive patients currently receiving ambiguous chemotherapy recommendations, this model’s granular risk quantification may reduce both overtreatment and therapeutic inertia (27, 28). This precision aligns with global initiatives to optimize chemotherapy stewardship through AI-powered clinical decision support systems.

While this study yielded promising results, it is crucial to recognize its limitations. Firstly, the external validation cohort consists only of BC patients from a single center. Therefore, AORSFM will require validation in additional centers in the future to further establish its reliability. Secondly, due to the limitations of the SEER database, information such as chemotherapy regimens, chemotherapy cycles, specific surgical-related indicators, and pre- and post-treatment laboratory tests were not included in the study. In the future, when validating and updating AORSFM in multicenter large-sample cohorts, the study will include these indicators to eliminate this limitation and enhance the model’s performance. Thirdly, molecular pathology features were not integrated into the model, which could have otherwise enhanced its predictive accuracy. Finally, the retrospective nature of data collection from SEER and a hospital resulted in instances of missing data. While this limitation might be offset by strict inclusion and exclusion criteria and a large sample size, prospective international multicenter studies are necessary to further validate the performance of the AORSFM.

## Conclusion

In summary, this study first externally validated the performance of the ODX nomogram in predicting the need for ACT. Then, the study developed the AORSFM for accurately predicting OS in BC patients who underwent radical surgery. AORSFM demonstrated stable and good predictive performance, calibration, and clinical net benefit across three cohorts. With its outstanding accuracy and reliability, AORSFM may serve as an effective tool for predicting OS in BC patients and guiding ACT after surgery.

## Data Availability

The data analyzed in this study is subject to the following licenses/restrictions: The protocol and statistical analysis methods used in the study can be requested directly from the corresponding author after approval. Requests to access these datasets should be directed to yangx1989@hotmail.com.
